# A simple parametric representation of the Hodgkin-Huxley model

**DOI:** 10.1371/journal.pone.0254152

**Published:** 2021-07-22

**Authors:** Alejandro Rodríguez-Collado, Cristina Rueda

**Affiliations:** Department of Statistics and Operations Research, Universidad de Valladolid, Valladolid, Spain; University of South Florida, UNITED STATES

## Abstract

The Hodgkin-Huxley model, decades after its first presentation, is still a reference model in neuroscience as it has successfully reproduced the electrophysiological activity of many organisms. The primary signal in the model represents the membrane potential of a neuron. A simple representation of this signal is presented in this paper. The new proposal is an adapted Frequency Modulated Möbius multicomponent model defined as a signal plus error model in which the signal is decomposed as a sum of waves. The main strengths of the method are the simple parametric formulation, the interpretability and flexibility of the parameters that describe and discriminate the waveforms, the estimators’ identifiability and accuracy, and the robustness against noise. The approach is validated with a broad simulation experiment of Hodgkin-Huxley signals and real data from squid giant axons. Interesting differences between simulated and real data emerge from the comparison of the parameter configurations. Furthermore, the potential of the FMM parameters to predict Hodgkin-Huxley model parameters is shown using different Machine Learning methods. Finally, promising contributions of the approach in Spike Sorting and cell-type classification are detailed.

## Introduction

Neuroscience is an interdisciplinary science that studies the cellular, functional, behavioral, evolutionary, computational, molecular, and medical aspects of the nervous system. Many specialists from different areas of knowledge, such as physicists, chemists, mathematicians, computer engineers, and psychologists, have contributed to the field. The mathematical approach is one of the most preferred ones, particularly in studying the electrophysiological activity between neurons. The signal that has received most of the attention is the neuron membrane potential, which is the difference in electric potential between the cell’s interior and exterior. This signal is composed of various Action Potential curves (APs). A single AP lasts a few milliseconds and consists of 3 stages: depolarization, repolarization, and hyperpolarization. For researchers, APs are of special importance: they are the informational unit between neurons, and their number and shape determine the morphological, functional, and genetic profile of the cell. For more detail see, [1–5].

According to their dynamic behavior, neurons can be classified as excitable (generate an individual AP) or oscillatory (generate repetitive APs). Neurons that do not generate APs are called non-excitable. Neuronal cells of a similar type habitually exhibit similar behaviors. For instance, cardiac myocytes are usually oscillatory, while cortical neurons are mostly excitable. Furthermore, oscillatory neurons have been sub-classified in this work by the observed number of APs. A signal where several APs are observed is known as a Spike Train.

The most broadly considered mathematical model for describing AP dynamics is the Hodgkin-Huxley (HH) model, presented in [6]. More than a half-century later, this model remains key in neuroscience due to its innovative concept of modeling neuronal dynamics as a system of Ordinary Differential Equations (ODE) and its accurate representation of the electrophysiological neuronal activity. Specifically, the neuron’s membrane potential is stated to behave like an electrical circuit with various currents associated with three types of ions: sodium (Na^+^), potassium (K^+^) and another which is a non-specific leak current, mainly due to the influx of chlorine (Cl^−^). However, the HH model lacks identifiability as various parameter configurations can lead to the same observed signal. In addition, the model is not robust as minor manipulations of the values of the parameters can change its output completely ([7, 8]).

Many models that have been developed afterward are either simplifications or extensions of the HH model. Some emulate the HH model as biophysically realistic, whereas others seek more simple models. Among the first group are the Hopfield model and the Van der Pol oscillator’s extensions, such as the FitzHugh-Nagumo model. In the second group, some popular choices are the family of leaky integrate-and-fire models and the Izhikevich model. Some basic references about these models are [9–13]. All the above are mechanistic models. The counterpart of the mechanistic models is the data-driven approach. Models based in data science, statistics and Machine Learning are in ever-rising popularity due to the increase in data availability and quality as noted in [14]. Our proposal can be framed within this class of models.

The Frequency Modulated Möbius (FMM) approach and others, such as the Fourier method, are encompassed in the amplitude modulated-frequency modulated decompositions. A general overview on these decompositions and time-frequency signal analysis can be found in [15, 16]. In particular, the FMM decomposition assumes a constant amplitude and a frequency that is modeled as a Möbius transformation. The monocomponent FMM model is presented in [17]. It shows how it accurately fits a wide variety of oscillatory patterns. The multicomponent FMM model is introduced in [18] and, in this, its potential in neuroscience is concisely demonstrated. Moreover, an exciting application for the automatic analysis of electrocardiograms is presented in [19].

This paper’s main goal is to show that the FMM model faithfully represents the AP signals waveforms derived from an HH model. To that end, a new FMM model is presented, denoted as FMM_ST_, where ST stands for Spike Train. It is an FMM multicomponent model with restrictions on the parameters. Expressly, the model assumes that the Spike Train is the concatenation of a fixed number of successive spikes with the same shape, each one described with a bi-component FMM.

The approach is validated with a broad simulation experiment and real data. In the first case, a total of 5000 HH signals, corresponding to a wide variety of parameter configurations, has been generated according to a factorial design for the most relevant HH parameters. It is shown how the FMM_ST_ model accurately predicts the simulated signals across all the parameter configurations. In the second case, signals from the Squid Giant Axon Membrane Potential (SGAMP) database, originally from [20], have been analyzed. These signals have been chosen because they inspired originally the HH model definition [6]. Interesting differences between the simulated and real data emerge from the comparison of the parameter configurations.

Furthermore, the simulated data is used to illustrate the potential of the FMM parameters to predict HH model parameters using different Machine Learning methods. It is shown how specific HH parameters, with relevant physiological interpretation, can be accurately predicted from the FMM parameters, which is important in real scenarios where the underlying HH model is unknown.

## Methods

### HH model

The presentation in this section follows those in [21, 22]. The notation for HH variables and parameters is slightly changed from the one used in these papers to avoid confusion with other terms introduced later in the paper.

The HH model is defined, see Definition 1 below, by a nonlinear ODE system for four state variables: the membrane potential (*X*) and the three opening probabilities of the ion gates (*m*, *n*, *h*). Furthermore, *X* depends on the input stimulus *I*(*t*) generated by other neurons’ post-synaptic currents. On their behalf, the variables *m*, *n* and *h* are referred to as voltage-gated channels as they depend on the membrane potential through the six ion gate transition functions denoted by *a*_*j*_ and *b*_*j*_, *j* ∈ {*m*, *n*, *h*}, as explained in [12].

**Definition 1**. Hodgkin-Huxley (HH) model.

$$\begin{array}{c} \left[\begin{array}{c} \frac{dX}{dt}\\ \frac{dm}{dt}\\ \frac{dn}{dt}\\ \frac{dh}{dt} \end{array}]=[\begin{array}{c} \frac{1}{C}\left[-g_{K}n^{4}\left(X-V_{K}\right)-g_{Na}m^{3}h\left(X-V_{Na}\right)-g_{L}\left(X-V_{L}\right)+I\left(t\right)\right]\\ \left(1-m\right)a_{m}\left(X\right)-mb_{m}\left(X\right)\\ \left(1-n\right)a_{n}\left(X\right)-nb_{n}\left(X\right)\\ \left(1-h\right)a_{h}\left(X\right)-hb_{h}\left(X\right) \end{array}\right] \end{array}$$

The first equation in Definition 1 depends on the parameters of the cell capacitance (*C*), the maximum conductances (*g*_*K*_, *g*_*Na*_, *g*_*L*_) and the equilibrium potentials (*V*_*K*_, *V*_*Na*_, *V*_*L*_) of the ionic currents. The six ion gate transition functions increase the number of parameters to more than twenty parameters. Hence, the parametric space of the model can be simplified by replacing the ion gate transition functions *a*_*m*_(*X*), *a*_*n*_(*X*), *a*_*h*_(*X*), *b*_*m*_(*X*), *b*_*n*_(*X*), and *b*_*h*_(*X*) with the constants ${\tilde{a}}_{m},{\tilde{a}}_{n},{\tilde{a}}_{h},{\tilde{b}}_{m},{\tilde{b}}_{n},$ and ${\tilde{b}}_{h}$, as done in [7, 5].

An interesting simplification of the HH parametric space is the pair (S,K) that has been recently considered in [7] and is defined as follows:

$$\begin{array}{c} S=\frac{g_{Na}}{g_{Na}+g_{K}}\;\;\;\;K=\frac{{\tilde{a}}_{n}+{\tilde{b}}_{m}}{{\tilde{a}}_{n}+{\tilde{b}}_{n}+{\tilde{a}}_{m}+{\tilde{b}}_{m}}. \end{array}$$ (1)

The aforementioned paper explains the properties of the (S,K) pair. In particular, they claim that the neuron’s excitability phenomenon is essentially bidimensional, being determined by the structure of the neuron as a cell and its ionic current kinetics. While S captures the neuron’s structural information, K represents the kinetics of the ionic gates. Moreover, (S,K) are less sensitive to slight changes in the signal than the primary HH parameters. However, such a drastic reduction in dimensions does not give a complete representation of the model.

### FMM approach

Let *t*_*i*_, *i* = 1, …, *n* denote the vector of observed time points and *X*(*t*_*i*_) the observed data, which in this paper is the potential difference across the neuron’s membrane. It is assumed that the time points are in [0, 2*π*). Otherwise, consider *t*′ ∈ [*t*_0_, *T* + *t*_0_] with *t*_0_ as the initial time value and *T* as the period. Transform the time points by $t=\frac{(t^{\prime }-t_{0})2\pi }{T}$.

Let, ***υ*** = (*A*, *α*, *β*, *ω*)′ be the four-dimensional parameters describing a monocomponent FMM signal, defined as the following *wave*: *W*(*t*, *υ*) = *A*cos(*ϕ*(*t*, *α*, *β*, *ω*)), where *A* is the amplitude and,

$$\begin{array}{c} \phi \left(t,\alpha ,\beta ,\omega \right)=\beta +2\arctan(\omega \tan\left(\frac{t-\alpha }{2}\right)) \end{array}$$ (2)

is the phase. Particularly, the phase is defined in terms of *α*, *β* and *ω*, parameters that determine the location, skewness and kurtosis respectively. More details about the parameters can be found in [17].

The FMM approach relies upon a signal plus error model, as follows:

$$\begin{array}{c} X\left(t_{i}\right)=M+\sum_{J=1}^{m}W\left(t_{i},\upsilon_{J}\right)+e\left(t_{i}\right),\;\;i=1,\ldots ,n; \end{array}$$ (3)

where the error term is assumed to be (*e*(*t*_1_), …, *e*(*t*_*n*_))′ ∼ *N*_*n*_(0, *σ*^2^ ***I***) and *M* is an intercept parameter. Note that this parameter does not represent the signal’s baseline level but its level at *t*_0_ minus the sum of waves values at *t*_0_.

The papers [19, 18] consider particular FMM models, show the broad type of signals that the model represents, provide properties, and interpret the parameters as well as detail the algorithm used to fit the models. In particular, in the second paper, its potential in neuroscience is concisely shown.

Depending on the application, the waves of the model represent different physiological processes. For instance, in the ECG case, the waves of the FMM model represent the five fundamental ECG upward and downward deflections, which are universally named P, QRS complex (a wave complex), and T.

The AP signals are modeled using two (or three in some cases) waves, the first being much more relevant than the rest. This wave, denominated dominant wave, identifies when the neuron spikes, allows the AP’s approximate reconstruction in the presence of noise, and the detection and identification of overlapping spikes. Moreover, theoretical properties are derived for the dominant wave. All of these is shown in [18].

**Fig 1:**
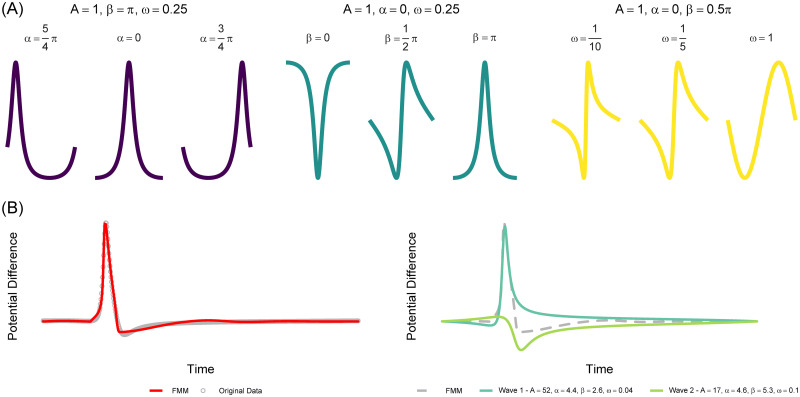
(**A**) FMM wave patterns for different parameter configurations. (**B**) Typical AP from an excitable neuron and corresponding fitted FMM model (left). Waves composing the FMM model (right).

Fig 1(A) shows FMM wave patterns by plotting *W*(*t*, *υ*) against *t* for different parameter configurations ***υ***. In Fig 1(B), (left) an AP signal from an excitable neuron (dots in grey) is represented along with the corresponding fitted FMM model (in red) whereas in Fig 1(B), (right), *W*(*t*, *υ*_*J*_), *J* = 1, 2, is plotted against *t*.

The FMM_ST_ model is a particular multicomponent FMM model, where restrictions on the parameters are imposed. The model is defined as follows:

**Definition 2**. FMM_ST_ Model

$$\begin{array}{c} X\left(t_{i}\right)=M+\sum_{S=1}^{s}W\left(t_{i},\upsilon_{S}^{A}\right)+W\left(t_{i}\upsilon_{S}^{B}\right)+e\left(t_{i}\right),\;\;i=1,\ldots ,n; \end{array}$$

where,

- *M* ∈ ℜ; $\upsilon_{S}^{A},\upsilon_{S}^{B}\in {ℜ}^{+}\times \left[0,2\pi \right]\times \left[0,2\pi \right]\times \left[0,1\right]$; *S* = 1, …, *s*,
- $A_{S}^{A}<A_{S}^{B}$; *S* = 1, …, *s*
- $\pi \leq \alpha_{1}^{A}\leq \alpha_{1}^{B}\leq \alpha_{2}^{A}\leq \alpha_{2}^{B}\ldots \leq \alpha_{S}^{A}\leq \alpha_{S}^{B}\leq \alpha_{1}^{A}\leq \pi$
- $\beta_{1}^{A}=\beta_{S}^{A}$ and $\beta_{1}^{B}=\beta_{S}^{B}$; *S* = 2, …, *s*.
- $\omega_{1}^{A}=\omega_{S}^{A}$ and $\omega_{1}^{B}=\omega_{S}^{B}$; *S* = 2, …, *s*.
- (*e*(*t*_1_), …, *e*(*t*_*n*_))′ ∼ *N*_*n*_(0, *σ*^2^ ***I***),

where *s* is the number of spikes that is assumed to be known and can be easily estimated with a naive method as is detailed in the estimation algorithm section.

The model also assumes that each spike is modelled with two waves with parameters ***υ***^*A*^ and ***υ***^*B*^, respectively. Other parameters of interest in practice are the distance between the *A* and *B* waves for a given AP, defined as $\cos(\alpha_{S}^{A}-\alpha_{S}^{B}),S=1,\ldots ,s$, and the distance between consecutive APs, $d_{S}^{AP}=1-\cos\left(\alpha_{S}^{A}-\alpha_{S+1}^{A}\right),S=1,\ldots ,s-1$. The inclusion of restrictions between the parameters is a specific feature of the FMM models, and their role is twofold. On the one hand, parameter identifiability is attained including the restriction *A* > 0 in the monocomponent case and, the restrictions between the *α*s and *A*s in the multicomponent case, in addition. According to that, the number of free parameters of FMM_ST_ model is 1 + 4*s* + 4. On the other hand, restrictions on the *ω*s and *β*s, which represent the equal spike shapes, provide physiologically interpretable solutions.

Furthermore, depending on the application at hand, additional restrictions may be imposed in order to use a simpler model. In particular, the following restrictions on the *A* parameters,

$$\begin{array}{c} A_{1}^{A}=A_{S}^{A}\;\text{and}\;A_{1}^{B}=A_{S}^{B};\;S=2,\ldots ,s \end{array}$$ (4)

are suitable in signals without APs generated at the beginning and/or end of the stimulus application, as these may exhibit different amplitudes than the rest of the APs. These restrictions have been considered in the SGAMP data analysis. In this case, the number of free parameters is reduced to 1 + 2*s* + 6.

Finally, in cases in which the distances between consecutive spikes are assumed to be constant, for instance in controlled experiments without stimulus changes, it would be fair to assume $d_{1}^{AB}=d_{S}^{AB};S=2,\ldots ,s$. Moreover, if the time lapse between the AP’s spike and the end of the hyperpolarization is constant, an additional set of restrictions may be imposed: $d_{1}^{AP}=d_{S}^{AP};S=2,\ldots ,s-1$. The number of free parameters in the first case is 1 + *s* + 8 whereas, in the second is 1 + 1 + 8.

#### Estimation algorithm

The implementation of FMM models in the programming language R, including applying the defined restrictions, is openly available in package FMM of the programming language R, first presented in [23]. It is assumed that the segments to be analyzed represent complete spikes, in particular, *X*(*t*_1_) ≃ *X*(*t*_*n*_). s is easily determined by a naive method based on a threshold as proposed [24]. This threshold is *k* = 2.5*σ*_*X*_, *σ*_*X*_ being the sample standard deviation of the observed data.

Occasionally, two different parameter configurations represent a given signal equally well. However, one is physiologically more plausible. In that case, additional restrictions on the parameters are needed to guarantee that the solution is the one expected. In the case of SGAMP signals, it is assumed that a spike has a prominent dominant wave which means that *A*^*A*^ − *A*^*B*^ > *C*, for a given threshold, *C*.

#### Validation measure

The goodness of fit of the model is measured with an *R*^2^ statistic, which is the proportion of the variance explained by a model out of the total variance, as follows:

$$\begin{array}{c} R^{2}=1-\frac{\sum_{i=1}^{n}{\left(X\left(t_{i}\right)-\hat{X}\left(t_{i}\right)\right)}^{2}}{\sum_{i=1}^{n}{\left(X\left(t_{i}\right)-\bar{X}\right)}^{2}} \end{array}$$ (5)

where $\hat{X}\left(t_{i}\right)$ represents the fitted value at *t*_*i*_, *i* = 1, …, *n*. In this paper, $R_{{\text{FMM}}_{\text{ST}}}^{2}$, $R_{{\text{FMM}}_{\text{ST}*}}^{2}$, $R_{{\text{FMM}}_{s*}}^{2}$ $R_{{\text{FMM}}_{s**}}^{2}$ refer to the *R*^2^ value for the FMM_ST_ model, the FMM_ST_ model with restrictions on the *A*s, the FMM_*s*_ model with restrictions on *β*s and *ω*s, and the FMM_s*_ model with restrictions on the *A*s, respectively.

#### Machine Learning Supervised methods

Several Machine Learning Supervised methods have been considered in the paper. At one end, the simple Linear Regression (LR) that serves as a benchmark approach. At the other extreme, the complex and “black box” Support Vector Machines of RBF Kernel (SVM) approach that has been proved to achieve excellent results in neuronal dynamics, as seen in [7, 13], among others. Random Forest (RF) and Gradient Boosting Machines (GBM) are complex methods that provide interpretable results between the underfitting LR and the overfitting SVM, which have also been considered. [25, 26] are essential references to learn about the procedures. The R packages [27–30], and the auxiliary package for learning procedures caret [31] have been used to implement the procedures.

#### Programming languages

The simulation experiment has been developed combining the programming languages Python and R, which are probably the most used programming languages in data science. Python is used for data acquisition and transformation, while R fits the FMM models. Several solutions have been studied for the coupling between them that could, at the same time, be computationally effective, robust, and simple. A basic outline on the matter is presented in [32]. While certain libraries provide tools for the coupling of the two languages, such as [33–35], these solutions are not sufficiently refined, and bash scripting was finally used.

## Results

### Simulation experiment design

In the first stage, Python simulates AP signals using a modified HH model implementation based on the one available in the Neurodynex package [21]. The original implementation offers several features, such as a detailed evolution of the voltage-gated variables *m*, *n*, *h*, or the application of input stimuli with different amplitude and shape. A modification was implemented to facilitate changes in the model parameters. The analyzed signal spans 60 ms. A short square input stimulus *I*, of just 1 ms, has been applied in the tenth ms of the simulation. See [36] to learn about the most used stimulus types. Five values for the strength of the stimulus have been selected, *I* = {0, 4.5, 7, 9.5, 12} *μA*, and 1000 experiments were conducted for each value of *I*, leading to a total of 5000 experiments. Different configurations of the most influential parameters have been considered according to a factorial experiment design. In every experiment, each of these parameters takes a random value from a set of preselected values. These values have been experimentally observed in nature, as described in [7] and detailed in Table 1.

**Table 1:** Parameters varied in the Hodgkin-Huxley simulations according to the designed factorial experiment design.

Structural Parameters	Kinetic Parameters
*C* = 1 *μ*F/cm^2^	${\tilde{a}}_{n}$ = {0.85, 0.95, 1.05, 1.15}
*V*_*Na*_ = 50 mV	${\tilde{b}}_{n}$ = {0.7, 0.85, 1, 1.15, 1.3}
*V*_*K*_ = −77 mV	${\tilde{a}}_{m}$ = {0.8, 0.9, 1, 1.1, 1.2, 1.3}
*V*_*L*_ = −54 mV	${\tilde{b}}_{m}$ = {0.7, 0.85, 1, 1.15, 1.3}
*g*_*Na*_ = {64, 92, 120, 148, 176, 204, 232, 260}ms/cm^2^	${\tilde{a}}_{h}$ = 1
*g*_*K*_ = {27, 30, 33, 36, 39, 42, 45, 48} ms/cm^2^	${\tilde{b}}_{h}$ = 1
*g*_*L*_ = {0.12, 0.215, 0.31, 0.405, 0.5} ms/cm^2^	

**Fig 2:**
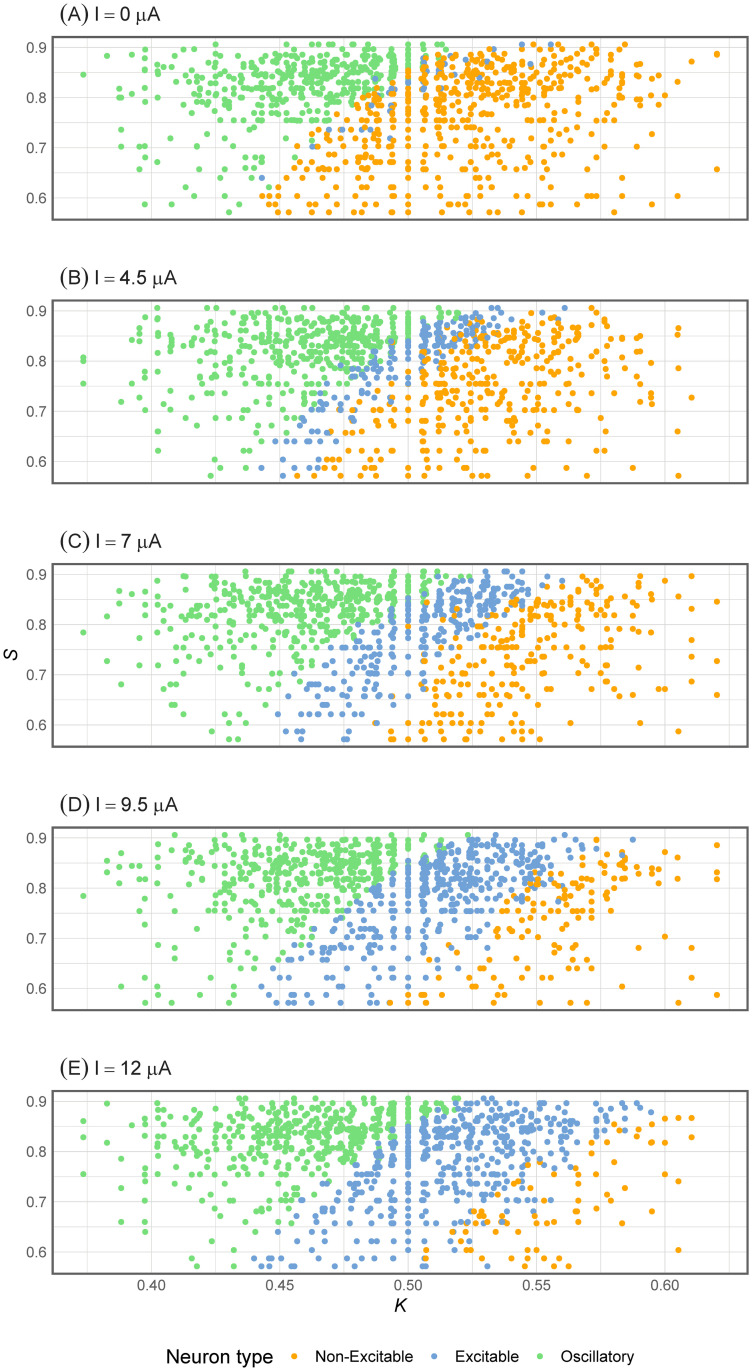
(S,K) values by stimulus amplitude and neuron type.

For the values in Table 1, (S,K) have been calculated with Eq (1) to be represented in Fig 2 across stimulus intensities by neuron type. While Fig 2(C) reproduces the one in [7], the rest of the planes generated by other stimulus intensity strengths are a novelty of this work, showing how the application of a stronger stimulus generates more excitable and oscillatory experiments.

In a second stage, using R, the signals are preprocessed to assure that complete APs are analyzed. We proceed as follows: the first AP is discarded if it occurs before the input stimulus has been applied; resting potential values are considered instead of the discarded data to get segments of equal length for all the trials. An analogous procedure is applied if the last AP is observed in the last 6 ms of the experiment. In the exceptional case, where only a pre-stimulus AP is observed, the original signal is analyzed. It is relevant to note that discarding the first and/or last AP in the experiments is not a limitation of the approach, as we are assuming equally-shaped APs.

In a third step, an FMM_ST_ model is fitted to the data using the FMM package.

### Simulation experiment results: FMM results

**Fig 3:**
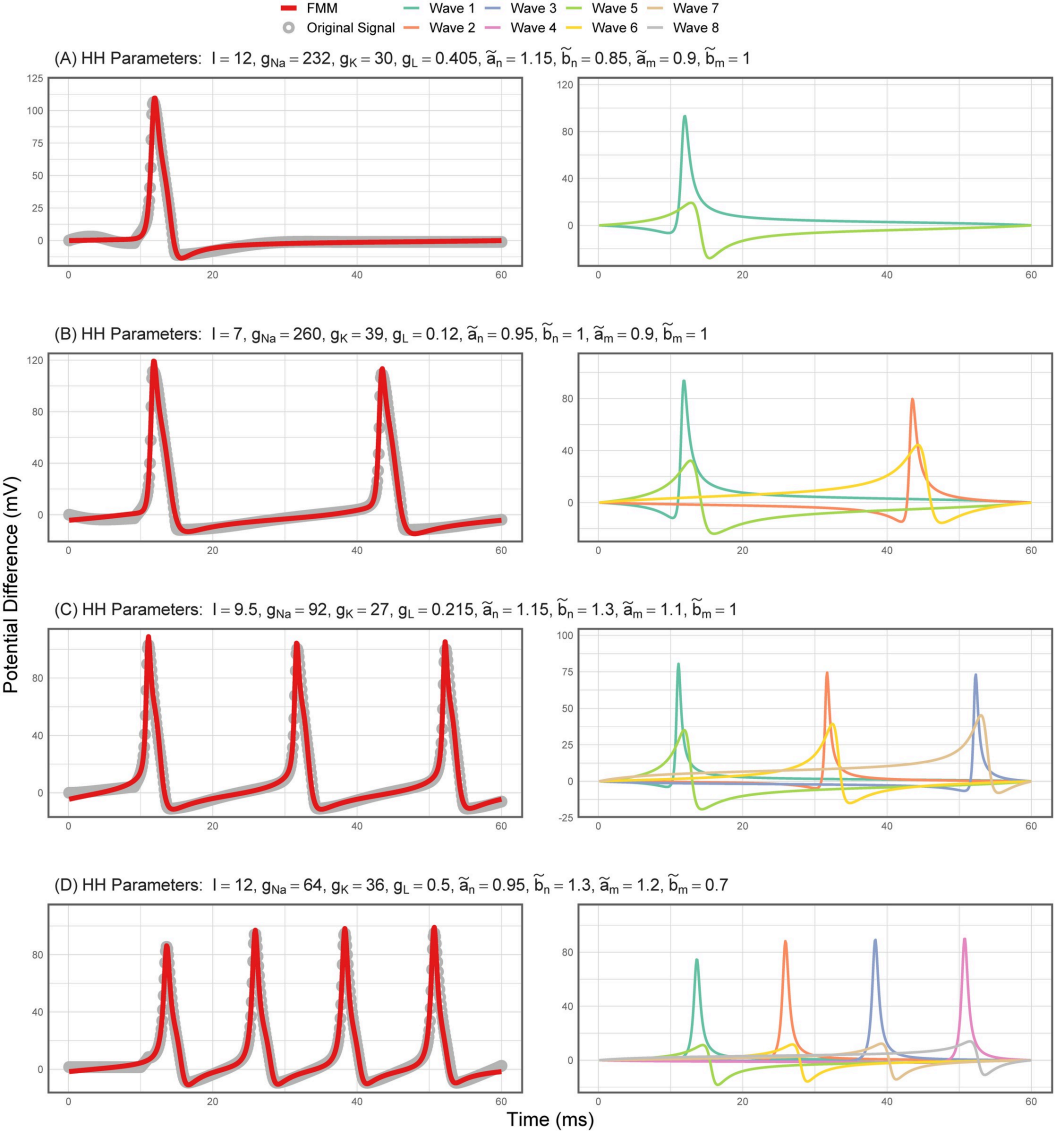
**Neuronal signals simulated with the HH model and the estimated FMM_ST_ signals in red (left)**. Waves of the fitted FMM_ST_ model (right), which have been enumerated according to relevance and order.

From the total 5000 simulated HH experiments, the 3613 with at least one AP have been analyzed using the FMM approach. In Fig 3, representative signals with one to four spikes are plotted along with the FMM_ST_ model predictions. There is a relevant dominant wave that represents the depolarization and repolarization; and a second wave that accounts for the hyperpolarization. A summary of the main statistics and parameter estimates are given in Table 2. The values in the table show the high prediction accuracy. In particular, the $R_{{\text{FMM}}_{s*}}^{2}$ global mean (standard deviation) is equal to 0.8527 (0.0579), and the $R_{{\text{FMM}}_{\text{ST}}}^{2}$ is equal to 0.9877 (0.0053). These values quantify what Fig 3 shows. Table 2 also shows interesting differences in parameter configurations between signals with a different number of APs.

**Table 2:** Means and standard deviations for *R*^2^ values and parameter estimators.

	Number of APs in the Signal						ALL
	1	2	3	4	5	6	
$R_{{\text{FMM}}_{s*}}^{2}$	0.817	0.817	0.857	0.881	0.886	0.902	0.853
	(0.061)	(0.089	(0.056)	(0.028)	(0.027)	(0.030)	(0.058)
$R_{{\text{FMM}}_{\text{ST}}}^{2}$	0.985	0.986	0.989	0.990	0.989	0.988	0.988
	(0.005)	(0.009)	(0.006)	(0.003)	(0.003)	(0.004)	(0.005)
**M**	39.211	80.448	96.346	117.824	156.227	183.459	89.650
	(3.936)	(22.861)	(24.920)	(23.591)	(22.320)	(19.165)	(45.465)
$A_{m}^{A}$	53.342	50.831	50.541	52.115	54.033	54.313	52.397
	(4.258)	(4.215)	(4.445)	(3.646)	(3.048)	(2.629)	(4.182)
$A_{m}^{B}$	16.0133	25.292	24.396	22.549	20.986	18.169	20.538
	(5.954)	(5.106)	(4.077)	(3.636)	(3.382)	(2.987)	(5.826)
*β*^**A**^	2.780	2.529	2.560	2.582	2.582	2.589	2.645
	(0.279)	(0.214)	(0.180)	(0.152)	(0.131)	(0.152)	(0.232)
cos(*β*^**B**^)	0.606	−0.142	−0.110	−0.059	−0.040	−0.029	0.162
	(0.347)	(0.297)	(0.232)	(0.203)	(0.224)	(0.243)	(0.424)
sin(*β*^**B**^)	−0.573	−0.941	−0.960	−0.977	−0.973	−0.970	−0.831
	(0.428)	(0.092)	(0.111)	(0.028)	(0.037)	(0.049)	(0.320)
*ω*^**A**^	0.041	0.030	0.029	0.030	0.030	0.030	0.033
	(0.005)	(0.006)	(0.005)	(0.004)	(0.004)	(0.005)	(0.007)
*ω*^**B**^	0.136	0.086	0.070	0.066	0.0689	0.076	0.092
	(0.066)	(0.044)	(0.020)	(0.012)	(0.012)	(0.016)	(0.052)
$d_{m}^{\text{AP}}$		1.515	1.488	1.125	0.814	0.636	0.784
		(0.767)	(0.335)	(0.126)	(0.068)	(0.021)	(0.643)
$d_{m}^{\text{AB}}$	0.039	0.034	0.029	0.032	0.036	0.041	0.034
	(0.023)	(0.071)	(0.012)	(0.011)	(0.014)	(0.016)	(0.020)

**Fig 4:**
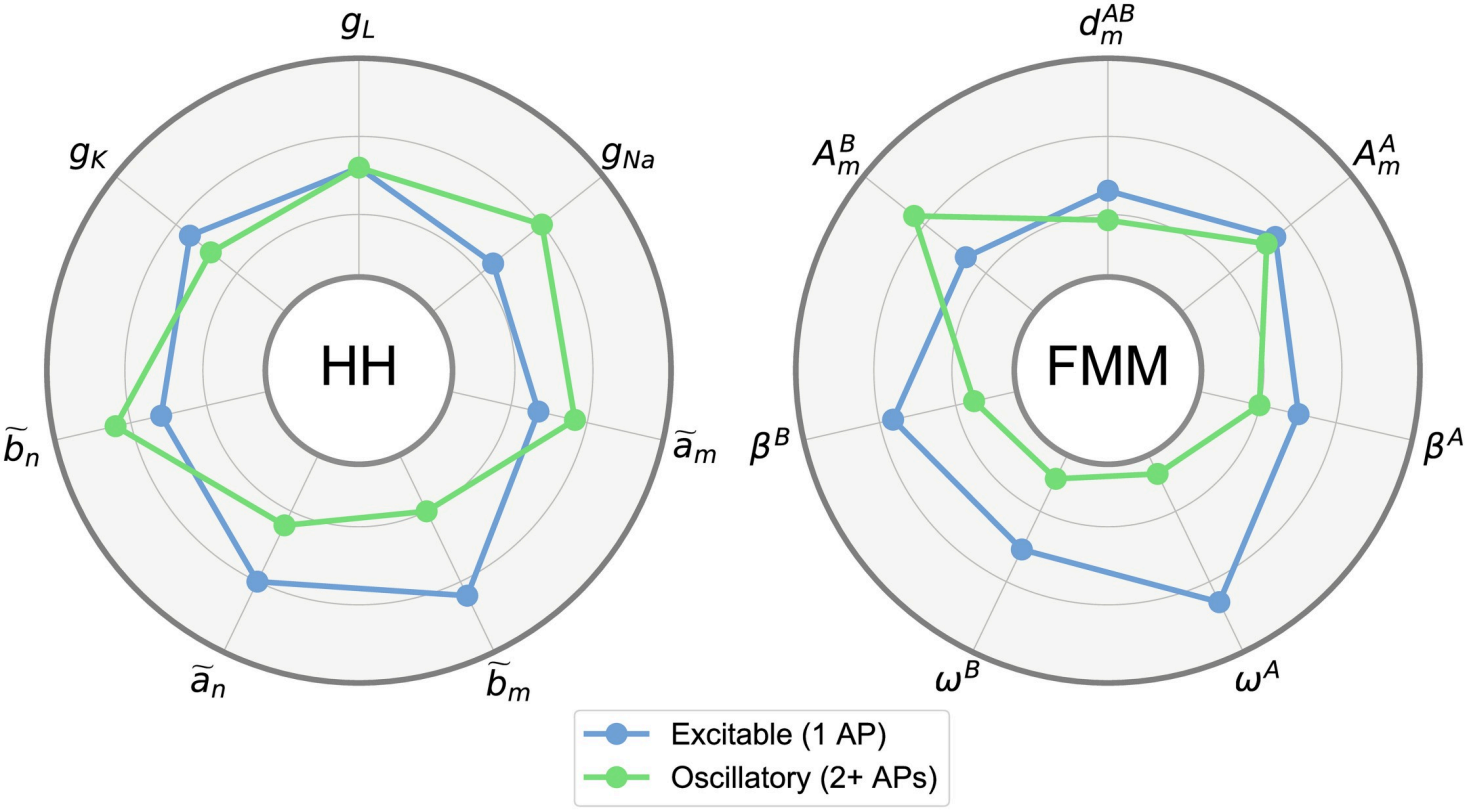
Median values of the HH parameters and FMM_ST_ parameters (defined in Table 3) by neuron type. The represented interval for each parameter is between the 10% and 90% percentiles.

In order to illustrate the parameter configurations differences between excitable and oscillatory neurons for HH and FMM models, two radar-plots have been represented in Fig 4. In the case of the HH parameters, which represent biochemical properties, oscillatory experiments have primarily higher values in *g*_*Na*_ and smaller values in ${\tilde{b}}_{m}$ and ${\tilde{a}}_{n}$. In terms of FMM parameters, which represent AP waveform features, oscillatory APs have nearer waves ($d_{m}^{AB}$) with smaller kurtosis and skewness (*ω*^*A*^, *ω*^*B*^, *β*^*A*^, *β*^*B*^) and a *B* wave with a higher amplitude ($A_{m}^{B}$).

### Simulation experiment results: Main HH parameters prediction

In this section, the potential of the FMM parameters to predict relevant parameters of the HH model is shown. Two different sets of predictors: *τ*^*A*^ and *τ*^*A*^ + *τ*^*B*^, defined in Table 3 and different Machine Learning Supervised procedures have been considered. Note that the LR and SVM approaches assume that the predictors are euclidean, but *β* is a circular parameter. Then, cos(*β*^*B*^) and sin(*β*^*B*^) are considered instead of *β*^*B*^. Furthermore, *β*^*A*^ is considered as euclidean as it takes values concentrated in a small arc. Other predictors, derived from those in *τ*^*A*^ + *τ*^*B*^, have been considered in the preliminary analysis but were eventually discarded using the principle of parsimony as only the results of LR improved lightly.

**Table 3:** Feature sets used in the prediction of the main HH parameters.

*τ*^*A*^		*τ*^*B*^	
*M*	Intercept parameter.		
$A_{m}^{A}$	Median of the amplitude $A_{S}^{A}$, *S* = 1, …, *s*.	$A_{m}^{B}$	Median of the amplitude $A_{S}^{B}$, *S* = 1, …, *s*.
*β*^*A*^	Skewness of the *A* waves.	$\beta^{B^{*}}$	Skewness of the *B* waves.
*ω*^*A*^	Kurtosis of the *A* waves.	*ω*^*B*^	Kurtosis of the *B* waves.
$d_{m}^{AP}$	Median of $d_{S}^{AP}$, *S* = 1, …, *s* − 1.	$d_{m}^{AB}$	Median of $d_{S}^{AB}$, *S* = 1, …, *s*
*s*	Number of APs in the signal.		

*: In linear procedures, cos(*β*^*B*^) and sin(*β*^*B*^) are used.

Variable selection methods have been used: a stepwise Akaike information criterion for LR, which selects the regression with the best trade-off between simplicity and goodness of fit, and caret’s recursive feature elimination [31] for the rest, which searched the minimal subset of variables that decreased at most 1.5% the goodness of fit. Only a single LR model reduces the used set of variables by discarding cos(*β*^*B*^).

Furthermore, ten-fold cross-validation is considered for comparative and validation purposes. The dataset is divided into ten equally sized splits. In ten iterations, nine of the subsets are used to train the procedure, while the tenth serves as a test as in [25, 37]. In addition, the Generalized Degrees of Freedom (GDFs) of the procedures have been calculated, as proposed in [38], to measure the underlying complexity.

Firstly, the prediction of the pair (S,K) is presented. Table 4 provides a summary of the results of the prediction procedures of S and K. It can be seen that the parameters associated with the second wave, *τ*^*B*^, significantly increase the prediction accuracy compared with that obtained using only parameters associated to wave *A*. Regarding the different procedures, at one end, the relatively bad results for LR provides evidence that the relation between the predictors and (S,K) is not linear. At the other end, SVM gives the most accurate prediction, with more than 95% and 94% of explained variance for S and K respectively. The RF and GBM results are between those for LR and SVM; while RF is comparable to GBM in terms of interpretability and complexity, the attained accuracy is less. However, although GBM procedures remain more complex and slightly less accurate than SVM, the predictions are interpretable.

**Table 4:** *R*^2^ and GDFs for the LR, RF, SVM and GBM procedures to predict S and K.

			**LR**	**RF**	**SVM**	**GBM**
S	**Predictors**: *τ*^***A***^	**R**^**2**^	0.6825	0.8421	0.8672	0.8576
		**GDF**	7.05	2053.13	301.28	1362.50
	**Predictors**: *τ*^***A***^ + *τ*^***B***^	**R**^**2**^	0.7933	0.9127	**0.9548**	0.9467
		**GDF**	12.21	2123.83	242.48	2699.63
			**LR**	**RF**	**SVM**	**GBM**
K	**Predictors**: *τ*^***A***^	**R**^**2**^	0.6542	0.8152	0.8446	0.8309
		**GDF**	6.74	2022.95	297.10	1766.07
	**Predictors**: *τ*^***A***^ + *τ*^***B***^	**R**^**2**^	0.7548	0.8877	**0.9413**	0.9350
		**GDF**	11.11	2113.74	381.73	2845.45

On the one hand, the most relevant predictors to explain S are *β*^***A***^ and *ω*^***A***^, which implies that S is related to the AP shape. In particular, AP signals from experiments with low S have more symmetrical *A* waves (*β*^***A***^ close to *π*). On the other hand, the most relevant predictors to explain K are $d_{m}^{AP}$, *M* and *s*, which recalls the strong association between K and the numbers of APs observed in Fig 2. Note that *s* is third in importance due to its discrete nature.

Predictive analysis for other HH parameters has also been performed. The results for sodium parameters are included in Table 5. Sodium parameters have been selected as they are more accurately predicted and are the neuron excitability engine, as authors such as [8, 39] claim. It is interesting to note that the ${\tilde{a}}_{m}$ and ${\tilde{b}}_{m}$ are directly related to the first and second FMM waves, respectively, while *g*_*Na*_ is related to both. The attained accuracy is not as high as for (S,K), which are more stable parameters.

**Table 5:** *R*^2^ values for LR, RF, SVM and GBM procedures to predict the sodium HH parameters.

		**LR**	**RF**	**SVM**	**GBM**
*g*_*Na*_	**Predictors**: *τ*^***A***^	0.5916	0.7207	0.7403	0.7309
	**Predictors**: *τ*^***A***^ + *τ*^***B***^	0.7106	0.7943	0.8276	0.8147
		**LR**	**RF**	**SVM**	**GBM**
${\tilde{a}}_{m}$	**Predictors**: *τ*^***A***^	0.3606	0.5801	0.6011	0.5909
	**Predictors**: *τ*^***A***^ + *τ*^***B***^	0.3760	0.7115	0.7810	0.7538
		**LR**	**RF**	**SVM**	**GBM**
${\tilde{b}}_{m}$	**Predictors**: *τ*^***A***^	0.2105	0.4013	0.3593	0.3901
	**Predictors**: *τ*^***A***^ + *τ*^***B***^	0.6521	0.8374	0.9220	0.9103

### Real data analysis

The SGAMP database, firstly used in [20] and publicly accessible at [40], contains single-unit neuronal recordings of North Atlantic squid (*Loligo pealei*) giant axons in response to stimulus currents. The database has been extensively used in works. Among the recent ones are [41, 42]. The experiments where the applied stimulus is a short square stimulus, the same as in the HH experimentation, have been selected for the analysis. Five signals have been extracted from 4 trials of 3 different axons, being the stimulus amplitude equal to *I* = 5 *μA* in the five cases. The length of the analyzed segment is also equal to that of the simulated HH signals, 60 ms, to facilitate comparisons.

Four different FMM models have been fitted to the signals: an FMM_s**_, FMM_s*_, FMM_ST_ and an FMM_ST*_. Moreover, it is assumed that *A*^*A*^ − *A*^*B*^ < *C*, where *C* is 0.70 times the maximum difference obtained in previous iterations of the algorithm. For comparative proposes, Fourier models with the same number of free parameters as the FMM models, denoted as FD_*a*_ where *a* is the number of harmonics, have also been fitted.

**Fig 5:**
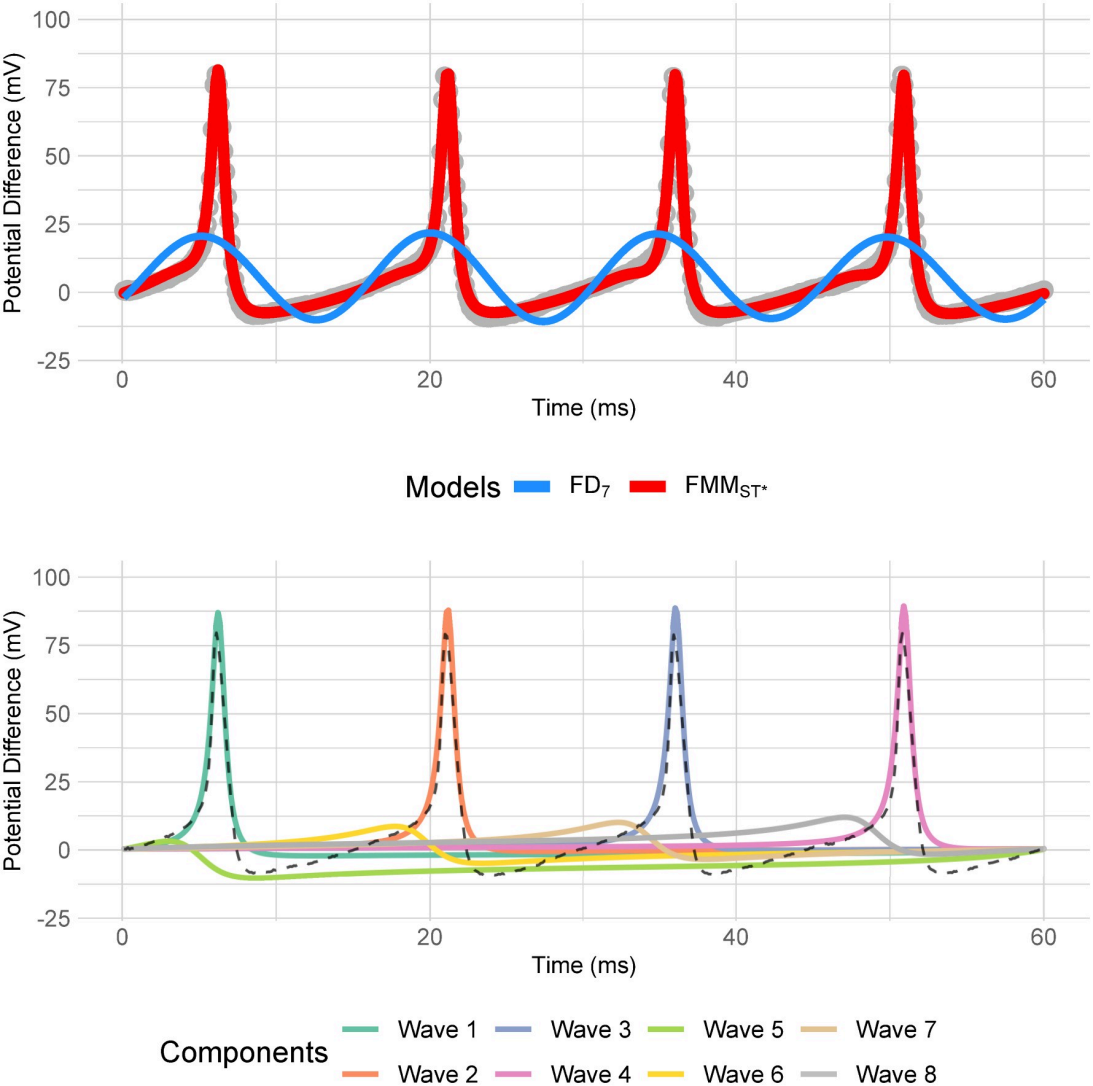
Neuronal APs from the SGAMP database (axon 1, trial 18, second short square stimulus) along with the fitted signals using an FMM_ST*_ signal (red) and an FD_7_ (blue). The waves of the fitted FMM_ST*_ model are illustrated at the bottom.

Fig 5 shows the FMM_ST*_ and the corresponding Fourier predictions for a representative signal. The *R*^2^ values and the number of free parameters are given in Table 6, for the five signals and eight models. The figures in the table show that the dominant wave explains much of the variability here, much more than it does in the HH experimentation. Furthermore, the models with restrictions in the *A* parameters are as accurate as the others without them. As such, the former models, being the simpler ones, are preferred due to the parsimony principle. Besides, the FD models make much less accurate predictions. Moreover, Table 7 gives the parameter values for the FMM_ST*_ model of the five signals. Compared to the values in Table 2, in particular to those corresponding to signals with 4 APs, some interesting differences can be highlighted. SGAMP APs have a more symmetrical shape (*β*^*A*^ values nearer to *π*) and a spike with a lower maximum voltage (smaller values of *A*^*A*^). Furthermore, shape differences in the repolarization and hyperpolarization are evidenced by *A*^*B*^ and *β*^*B*^.

**Table 6:** *R*^2^ values of the different FMM and FD models for the signals extracted from the SGAMP database.

	N° of parameters	Experiment IDs				
		a1t18_1	a1t18_2	a1t22	a3t03	a4t13
**FMM**_s**_	8	0.9435	0.9457	0.9472	0.9512	0.9324
**FMM**_s*_	11	0.9485	0.9511	0.9476	0.9566	0.9429
**FMM**_ST*_	15	0.9862	0.9848	0.9880	0.9936	0.9910
**FMM**_**ST**_	21	0.9865	0.9852	0.9882	0.9937	0.9910
**FD**_**4**_	9	0.3635	0.3609	0.3587	0.4080	0.2809
**FD**_**5**_	11	0.3648	0.3622	0.3594	0.4141	0.2865
**FD**_**7**_	15	0.3687	0.3636	0.3614	0.4305	0.3056
**FD**_**10**_	21	0.5676	0.5809	0.5586	0.6099	0.4695

**Table 7:** Parameter estimators of the FMM_ST*_ models for the SGAMP signals.

	Experiment IDs					Mean
	a1t18_1	a1t18_2	a1t22	a3t03	a4t13	
**M**	173.617	179.144	115.622	158.667	184.105	162.231
**A**^**A**^	44.955	44.472	37.352	43.089	46.284	43.230
**A**^**B**^	6.893	6.770	13.989	6.478	27.524	12.331
*β*^**A**^	3.426	3.311	3.933	3.572	3.634	3.575
cos(*β*^**B**^)	−0.368	−0.387	−0.214	−0.428	−0.221	−0.324
sin(*β*^**B**^)	−0.930	−0.922	0.977	−0.903	0.975	−0.161
*ω*^**A**^	0.028	0.028	0.030	0.027	0.027	0.028
*ω*^**B**^	0.101	0.138	0.008	0.097	0.005	0.070
$d_{m}^{\text{AP}}$	1.016	0.985	0.987	1.033	1.049	1.014
$d_{m}^{\text{AB}}$	0.021	0.020	0.002	0.020	0.002	0.013

### Parameter configurations comparison between simulated and real data

**Fig 6:**
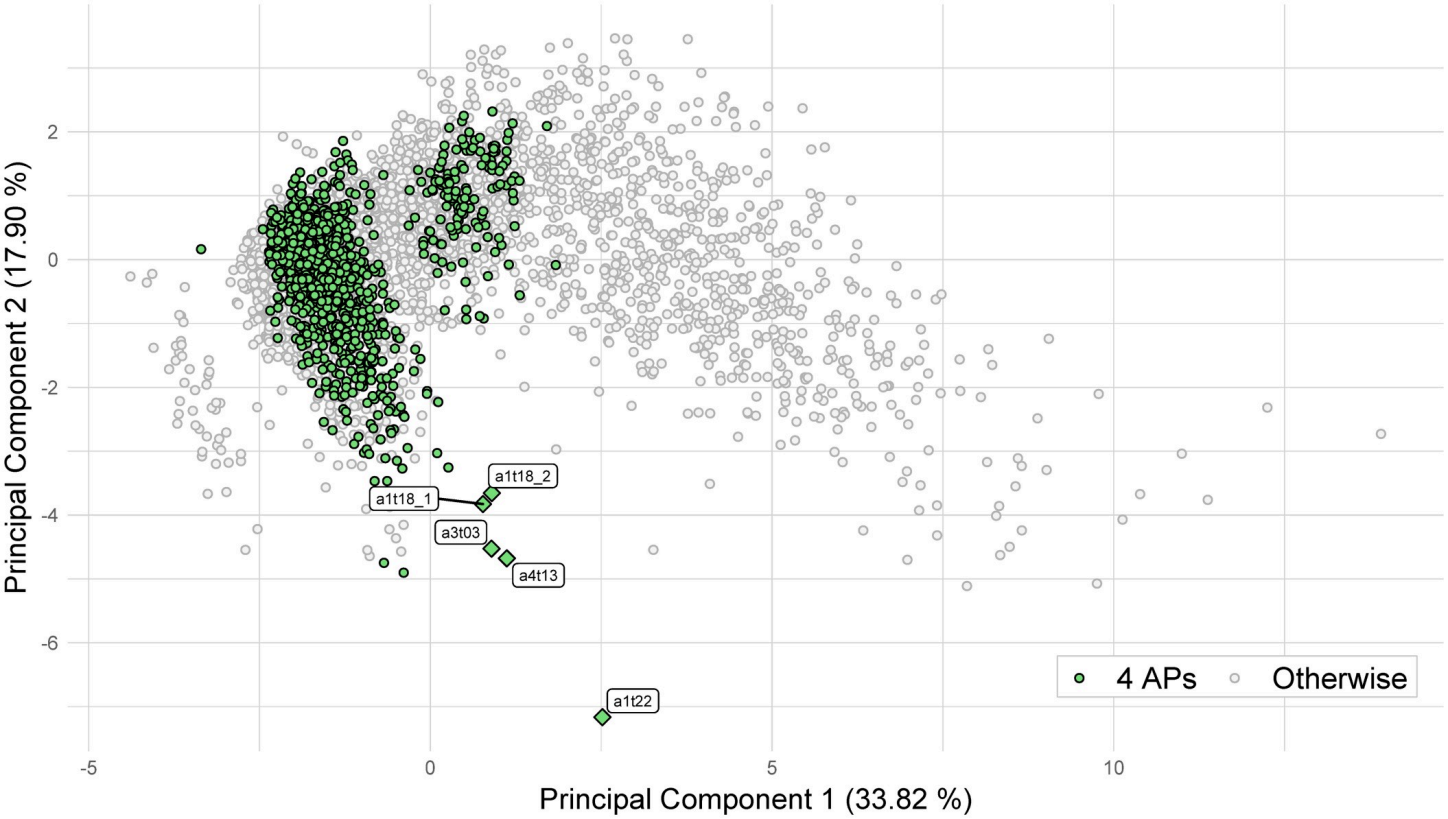
Plot of the first two principal components of *τ*^*A*^ + *τ*^*B*^ of the HH experiments (circular points), with experiments with 4 APs highlighted, and projections of the SGAMP experiments (rhombus points).

In order to facilitate the parameter configurations comparison between real data and HH simulations, a principal component analysis has been performed with the simulated data. Furthermore, the corresponding projections of the SGAMP parameter configurations into this plane have been obtained. The first two principal components are plotted in Fig 6, where circular points represent the simulated signals and the rhombus points represent the real data projections. The Figure clearly illustrates the differences between the parameter configurations of the simulated and real data, as rhombus points are far from the main cloud of circular points.

At first glance, these differences could be attributed to the simplifications of the HH experimentation done. However, in our opinion, this is not a plausible explanation. Specifically, the intensity and shape of the stimulus being fixed does not affect the AP shape modelled by the FMM parameters, as authors like [4, 43] state. The other simplifications in the experiment design are minor. Furthermore, a simpler FMM model is adequate to analyze the real signals accurately as Table 6 shows. All of these comments evidence that the model underlying SGAMP signals is not an HH but a simpler one, such as FitzHugh-Nagumo (see [9]).

## Discussion

In this paper, the FMM_ST_ model has been presented, and its potential to describe the waveforms of simulated and real AP signals has been proved. It is shown that the squid giant axon signals exhibit simpler waveforms and are faithfully described with a simpler model than the simulated HH signals. Moreover, the excellent behavior of the FMM model to predict HH simulated and real signals provides pieces of evidence that other neuronal dynamics models could also be represented using the new approach, in which case, the differences between models would be articulated through the differences in the parameter configurations.

In addition to many interesting theoretical properties, the FMM approach is a flexible methodology from an applied point of view. When a single AP is analyzed, the parameters are able to describe a wide variety of spikes morphologies and, in particular, to discriminate neuron types. When multiple spikes are analyzed, restrictions between the parameters can be included to provide physiologically interpretable solutions and reduce the model’s complexity.

Many open problems in electrophysiological neuroscience can benefit from the FMM methodology presented in this paper. In particular, it can significantly contribute to solving problems where the AP characterization is needed, such as Spike Sorting and cell-type classification. Spike Sorting implies grouping spikes into clusters corresponding to different neurons based on the similarity of their shapes. Cell-type classification deals with the definition of hierarchical taxonomies of cells based on different sets of morphological, genetic and/or electrophysiological features.

Spike Sorting and cell-type classification are two of the most critical data analysis problems in neuroscience and have received a lot of attention in the literature. Some interesting references about Spike Sorting are [24, 44, 45], whereas [2, 13, 46] address cell-type classification. Besides, it is worth mentioning other more complex related problems such as multi-channel Spike Sorting and the function identification of neurons in brain circuits. These questions that are tackled in [47–49].

Furthermore, the FMM approach, based on a simple parametric model, is opposite of ‘black box’ methods, which are becoming popular in neuroscience and other areas, and provides simple solutions to different questions. A particular case is the problem of denoising neuronal signals, which is essential when recordings are made *in vivo* because often low temporal resolution and noisy data are recorded in this scenario. Neuronal networks are the predominant methodology to solve this task (see [50, 51]); however, the FMM model is a signal plus error and the denoising is included in the estimation step.

Two different lines of work could be defined for the future. On the one hand, from a theoretical perspective, a first question to solve is the implementation of restrictions with the form $d_{1}^{AB}=d_{S}^{AB}$; *S* = 2, …, *s*. They are of interest to analyze signals with equal distances between waves in the APs, because reducing the number of parameters to be estimated is important when large or noisy Spike Trains are analyzed. On the other hand, from an applied perspective, many other AP real signals must be analyzed, and the questions of spike and cell classification and clustering may be addressed. The approach’s potential is difficult to calibrate as many aspects remain to be researched and exploited.

Finally, a limitation of our study is that the input stimulus’s timing and shape have been fixed. The influence of the stimulus type could be analyzed using the FMM approach. The *α* parameters are related to the firing times, and the shape parameters could be useful to detect circumstances where the shape of the APs is independent of the stimulus, as [4] suggests, and circumstances where changes happen, such as the observation of incomplete spikes. However, the question is tricky and deserves further research.

## References

[1] Mensi S, Naud R, Pozzorini C, Avermann M, Petersen CCH, Gerstner W. Parameter extraction and classification of three cortical neuron types reveals two distinct adaptation mechanisms. Journal of Neurophysiology. 2012;107(6):1756–1775. doi: 10.1152/jn.00408.2011 22157113

[2] Zeng H, Sanes J. Neuronal cell-type classification: Challenges, opportunities and the path forward. Nature Reviews Neuroscience. 2017;18. doi: 10.1038/nrn.2017.85 28775344

[3] Trainito C, von Nicolai C, Miller EK, Siegel M. Extracellular spike waveform dissociates four functionally distinct cell classes in primate cortex. Current Biology. 2019;29(18):2973–2982. doi: 10.1016/j.cub.2019.07.051 31447374

[4] Raghavan M, Fee D, Barkhaus PE. Generation and propagation of the action potential. In: Handbook of clinical neurology. vol. 160. Elsevier; 2019. p. 3–22. 31277855 10.1016/B978-0-444-64032-1.00001-1

[5] Ori H, Hazan H, Marder E, Marom S. Dynamic clamp constructed phase diagram for the Hodgkin and Huxley model of excitability. Proceedings of the National Academy of Sciences. 2020;117(7):3575–3582. doi: 10.1073/pnas.1916514117 32024761 PMC7035484

[6] Hodgkin AL, Huxley AF. A quantitative description of membrane current and its application to conduction and excitation in nerve. The Journal of Physiology. 1952;117(4):500–544. doi: 10.1113/jphysiol.1952.sp004764 12991237 PMC1392413

[7] Ori H, Marder E, Marom S. Cellular function given parametric variation in the Hodgkin and Huxley model of excitability. Proceedings of the National Academy of Sciences. 2018;115(35):E8211–E8218. doi: 10.1073/pnas.1808552115 30111538 PMC6126753

[8] Marom S. Emergence and maintenance of excitability: kinetics over structure. Current Opinion in Neurobiology. 2016;40:66—71. doi: 10.1016/j.conb.2016.06.013 27400289

[9] Fitzhugh R. Impulses and Physiological States in Theoretical Models of Nerve Membrane. Biophysical Journal. 1961;1(6):445–466. doi: 10.1016/s0006-3495(61)86902-6 19431309 PMC1366333

[10] Abbott LF, Kepler TB. Model neurons: From Hodgkin-Huxley to Hopfield. In: Statistical Mechanics of Neural Networks. Berlin, Heidelberg: Springer Berlin Heidelberg; 1990. p. 5–18.

[11] Izhikevich EM. Simple model of spiking neurons. IEEE Transactions on Neural Networks. 2003;14(6):1569–1572. doi: 10.1109/TNN.2003.820440 18244602

[12] Ermentrout B, Terman D. The Mathematical Foundations of Neuroscience. 1st ed. Springer; 2010.

[13] Teeter C, Iyer R, Menon V, Gouwens N, Feng D, Berg J, et al. Generalized leaky integrate-and-fire models classify multiple neuron types. Nature Communications. 2018;9(1):1–15. doi: 10.1038/s41467-017-02717-4 29459723 PMC5818568

[14] Brunton BW, Beyeler M. Data-driven models in human neuroscience and neuroengineering. Current Opinion in Neurobiology. 2019;58:21—29. doi: 10.1016/j.conb.2019.06.008 31325670

[15] Picinbono B. On instantaneous amplitude and phase of signals. IEEE Transactions on Signal Processing. 1997;45(3):552–560. doi: 10.1109/78.558469

[16] Boashash B. Time-Frequency Signal Analysis and Processing: A Comprehensive Reference. 1st ed. Academic Press; 2003.

[17] Rueda C, Larriba Y, Peddada SD. Frequency Modulated Möbius Model Accurately Predicts Rhythmic Signals in Biological and Physical Sciences. Scientific Reports. 2019;9(1):1–10. doi: 10.1038/s41598-019-54569-1 31822685 PMC6904729

[18] Rueda C, Rodríguez-Collado A, Larriba Y. A novel wave decomposition for oscillatory signals. IEEE Transactions on Signal Processing. 2021;69:960–972. doi: 10.1109/TSP.2021.3051428

[19] Rueda C, Larriba Y, Lamela A. The hidden waves in the ECG uncovered revealing a sound automated interpretation method. Scientific Reports. 2021;11:3724. doi: 10.1038/s41598-021-82520-w 33580164 PMC7881027

[20] Paydarfar D, Forger DB, Clay JR. Noisy Inputs and the Induction of On–Off Switching Behavior in a Neuronal Pacemaker. Journal of Neurophysiology. 2006;96(6):3338–3348. doi: 10.1152/jn.00486.2006 16956993

[21] Gerstner W, Kistler WM, Naud R, Paninski L. Neuronal Dynamics: From Single Neurons to Networks and Models of Cognition. 1st ed. New York, NY, USA: Cambridge University Press; 2014.

[22] Estumano D, Orlande HR, Colaço M, Ritto T, Diaz J, Dulikravich G. Bayesian Estimation of Parameters in Hodgkin-Huxley’s Model of Biomedical Electric Signals. In: 2nd International Symposium on Uncertainty Quantification and Stochastic Modeling; 2014. p. 1–11.

[23] Fernández I, Rodríguez-Collado A, Larriba Y, Lamela A, Canedo C, Rueda C. FMM: An R package for modeling rhythmic patterns in oscillatory systems. arXiv:2105.10168 [Preprint]; 2021. Available from: https://arxiv.org/abs/2105.10168.

[24] Rey HG, Pedreira C, Quiroga R. Past, present and future of spike sorting techniques. Brain Research Bulletin. 2015;119:106–117. doi: 10.1016/j.brainresbull.2015.04.007 25931392 PMC4674014

[25] Hastie T, Tibshirani R, Jerome F. The Elements of Statistical Learning: Data Mining, Inference, and Prediction. 2nd ed. Springer; 2009.

[26] Izenman AJ. Modern Multivariate Statistical Techniques: Regression, Classification, and Manifold Learning. 1st ed. Springer Publishing Company, Incorporated; 2008.

[27] R Core Team. Package ‘stats’ in R: A Language and Environment for Statistical Computing; 2013. Available from: http://www.R-project.org/.

[28] Breiman L, Cutler A, Liaw A, Wiener M. Package ‘randomForest’ Manual. CRAN; 2018.

[29] Karatzoglou A, Smola A, Hornik K, Zeileis A. kernlab—An S4 Package for Kernel Methods in R. Journal of Statistical Software. 2004;11(9):1–20. doi: 10.18637/jss.v011.i09

[30] Ridgeway G. Generalized Boosted Models: A Guide to the GBM Package. Compute. 2005;1:1–12.

[31] Kuhn M. Package ‘caret’ Manual. New London: CRAN; 2018.

[32] Pandey P. From ‘R vs Python’ to ‘R and Python’. Towards Data Science; 2019. Available from: https://towardsdatascience.com/from-r-vs-python-to-r-and-python-aa25db33ce17.

[33] Gautier L. rpy2—R in Python; 2019. Available from: https://rpy2.bitbucket.io/.

[34] Curtis V. RWinOut; 2017. Available from: https://github.com/vitorcurtis/RWinOut.

[35] Xia XQ, McClelland M, Wang Y. PypeR, A Python Package for Using R in Python. Journal of Statistical Software, Code Snippets. 2010;35(2):1–8.

[36] Allen Brain Institute. Allen Cell Types Database—Electrophysiology. Technical report; 2015. Available from: http://help.brain-map.org/download/attachments/8323525/CellTypes_Ephys_Overview.pdf?version=2&modificationDate=1508180425883&api=v2.

[37] Kubat M. An Introduction to Machine Learning. 1st ed. Springer Publishing Company, Incorporated; 2015.

[38] Ye J. On Measuring and Correcting the Effects of Data Mining and Model Selection. Journal of The American Statistical Association. 1998;93:120–131. doi: 10.1080/01621459.1998.10474094

[39] Silva J. Slow Inactivation of Na+ Channels. In: Voltage Gated Sodium Channels; 2014. p. 33–49. 10.1007/978-3-642-41588-3_3 24737231

[40] Goldberger AL, Amaral LAN, Glass L, Hausdorff JM, Ivanov PC, Mark RG, et al. PhysioBank, PhysioToolkit, and PhysioNet: Components of a New Research Resource for Complex Physiologic Signals. Circulation. 2000;101(23):e215–e220. doi: 10.1161/01.CIR.101.23.e215 10851218

[41] Forger D, Paydarfar D, Clay J. Optimal Stimulus Shapes for Neuronal Excitation. PLoS Computational Biology. 2011;7:e1002089. doi: 10.1371/journal.pcbi.1002089 21760759 PMC3131391

[42] Clay J, Forger D, Paydarfar D. Ionic Mechanism Underlying Optimal Stimuli for Neuronal Excitation: Role of Na+ Channel Inactivation. PloS One. 2012;7:e45983. doi: 10.1371/journal.pone.0045983 23049913 PMC3458826

[43] Kass RE, Amari SI, Arai K, Brown EN, Diekman CO, Diesmann M, et al. Computational Neuroscience: Mathematical and Statistical Perspectives. Annual Review of Statistics and Its Application. 2018;5(1):183–214. doi: 10.1146/annurev-statistics-041715-033733 30976604 PMC6454918

[44] Caro-Martín CR, Delgado-García JM, Gruart A, Sánchez-Campusano R. Spike sorting based on shape, phase, and distribution features, and K-TOPS clustering with validity and error indices. Scientific Reports. 2018;8(1):1–28. doi: 10.1038/s41598-018-35491-4 30542106 PMC6290782

[45] Souza BC, Lopes-dos Santos V, Bacelo J, Tort AB. Spike sorting with Gaussian mixture models. Scientific Reports. 2019;9(1):1–14. doi: 10.1038/s41598-019-39986-6 30842459 PMC6403234

[46] Ghaderi P, Marateb H, Safari MS. Electrophysiological Profiling of Neocortical Neural Subtypes: A Semi-Supervised Method Applied to in vivo Whole-Cell Patch-Clamp Data. Frontiers in Neuroscience. 2018;12. doi: 10.3389/fnins.2018.00823 30542256 PMC6277855

[47] Mannal N, Kleiner K, Fauler M, Dougalis A, Poetschke C, Liss B. Multi-Electrode Array Analysis Identifies Complex Dopamine Responses and Glucose Sensing Properties of Substantia Nigra Neurons in Mouse Brain Slices. Frontiers in Synaptic Neuroscience. 2021;13:1. doi: 10.3389/fnsyn.2021.635050 33716704 PMC7952765

[48] Bashford J, Mills K, Shaw C. The evolving role of surface electromyography in amyotrophic lateral sclerosis: A systematic review. Clinical Neurophysiology. 2020;131(4):942–950. doi: 10.1016/j.clinph.2019.12.007 32044239 PMC7083223

[49] Chen Y, Rommelfanger NJ, Mahdi AI, Wu X, Keene ST, Obaid A, et al. How is flexible electronics advancing neuroscience research? Biomaterials. 2021;268:120559. doi: 10.1016/j.biomaterials.2020.120559 33310538 PMC7856293

[50] Lecoq J, Oliver M, Siegle JH, Orlova N, Koch C. Removing independent noise in systems neuroscience data using DeepInterpolation. BioRxiv 2020.10.15.341602 [Preprint]; 2020. Available from: https://www.biorxiv.org/content/10.1101/2020.10.15.341602v2. 10.1038/s41592-021-01285-2 PMC8833814 34650233

[51] Sebastian J, Sur M, Murthy HA, Magimai-Doss M. Signal-to-signal neural networks for improved spike estimation from calcium imaging data. PLOS Computational Biology. 2021;17(3):1–19. doi: 10.1371/journal.pcbi.1007921 33647015 PMC7951974

